# Microspheres present comparable efficacy and safety profiles compared with polyvinyl alcohol for bronchial artery embolization treatment in hemoptysis patients

**DOI:** 10.1186/s12967-021-02947-7

**Published:** 2021-10-11

**Authors:** Zhigang Fu, Xun Li, Fei Cai, Yinpeng Yuan, Xiaolin Zhang, Jingxia Qin, Yonghui Liang

**Affiliations:** 1 Department of Radiology, Yichang Central People’s Hospital, First College of Clinical Medical Science, China Three Gorges University, 183 Yiling Road, Yichang, 443003 China; 2 Department of Radiology, People’s Hospital of Dangyang country, Dangyang, 444100 China

**Keywords:** Hemoptysis, Microspheres, Polyvinyl alcohol, Bronchial artery embolization, Hemoptysis recurrence, Mortality

## Abstract

**Background:**

The present study aimed to compare the efficacy and safety profiles of microspheres versus (vs.) polyvinyl alcohol (PVA) for bronchial artery embolization (BAE) treatment in patients with hemoptysis.

**Methods:**

Totally, 152 patients with hemoptysis who were about to receive BAE treatment were consecutively enrolled and divided into microspheres group (N = 62) and PVA group (N = 90). Technical success and clinical success were assessed after BAE procedure, and the hemoptysis-recurrence status, survival status and adverse events were recorded during follow-up.

**Results:**

Technical success rates were both 100% in microspheres group and PVA group; clinical success rate (96.8% vs. 100.0%, *P* = 0.165), 6-month (9.7% vs. 7.8%, *P* = 0.681) and 1-year (9.7% vs. 8.9%, *P* = 0.869) hemoptysis recurrence rate, 6-month (4.8% vs. 2.2%, *P* = 0.374) and 1-year (4.8% vs. 3.3%, *P* = 0.639) mortality were similar between microspheres group and PVA group. Furthermore, hemoptysis-free survival (*P* = 0.488) and overall survival (*P* = 0.321) were of no difference between two groups. In addition, all adverse events were mild, and there was no difference of adverse events between two groups (all *P* > 0.05). These data were validated by further multivariate regression analysis.

**Conclusions:**

Microspheres present comparable efficacy and safety profiles compared with PVA for the BAE treatment in patients with hemoptysis, providing evidence for embolic agent selection.

**Supplementary Information:**

The online version contains supplementary material available at 10.1186/s12967-021-02947-7.

## Background

Hemoptysis is the expectoration of blood originated from the respiratory tract, and is a clinical manifestation of various pulmonary or tracheobronchial diseases, including bronchiectasis, acute respiratory tract infections, asthma, chronic obstructive pulmonary diseases, and malignancy [1, 2]. Hemoptysis is regarded as a serious medical emergency, since hemorrhage can flood into airways, leading to the airway obstruction, impairment of ventilation, blood transfusion, and further the occurrence of asphyxia as well as cardiovascular collapse [3]. Even with the existing treatment strategy (including surgery, bronchial artery embolization (BAE), etc.), patients with moderate-to-massive hemoptysis still suffer from relative high risk of recurrence and morality [4–6]. Hence, development of effective and safe treatment strategies is essential to control bleeding and limit the spread of hemorrhage promptly, further guaranteeing the survival in patients with hemoptysis.

Bronchial artery embolization is an effective and minimally invasive palliative therapeutic approach for the management of hemoptysis, and presents approximately 90% immediate success rate for short-term hemoptysis control [7, 8]. The procedures of BAE include the cannulation placement in the targeted bronchial artery and embolization with various embolic agents, and polyvinyl alcohol (PVA) is one of the most extensively used embolic agents [7, 9]. Mechanically, PVA, as a non-biodegradable and biocompatible polymer, is compressed and then introduced into bloodstream, further swelling, and occluding a vessel for the BAE purpose [7]. The efficacy and safety of BAE with PVA in controlling massive hemoptysis has been illustrated, however, PVA still exhibits disadvantage of tendency to aggregate, which may increase the risk of proximal occlusion and long-term hemoptysis recurrence rate [7, 9–12]. Recently, microspheres has gained attention for hemoptysis control as a novel embolic agent in China. Compared with PVA, microspheres present less risk of aggregation, furthermore, it also displays various advantages, such as calibrated size, resistance to aggregate, satisfied elasticity [7, 13–15]. Microspheres has been revealed to be effective and safe in treating hyper-vascular tumor, however, its efficacy and safety has not been explored in hemoptysis yet [16, 17].

Therefore, we conducted the present study to compare the efficacy and safety profiles of microspheres versus (vs.) PVA for BAE treatment in patients with hemoptysis, which helped to provide evidence for embolic agent selection.

## Methods

### Patients

From June 2017 to May 2020, a total of 152 patients with hemoptysis and schedule to receive BAE treatment in our hospital were consecutively enrolled in this study. The inclusion criteria were (1) confirmed as hemoptysis, which was defined as airway bleeding with estimated hemoptysis volume > 20 mL in each event [18]; (2) age ≥ 18 years old, and (3) about to receive BAE treatment using polyvinyl alcohol (PVA) or microspheres. The exclusion criteria were (1) history of BAE for hemoptysis, (2) severe abnormality of cardio-pulmonary function or hepatorenal function, (3) contraindication to BAE, and (4) women in pregnancy or lactation. In addition, lung cancer patients with hemoptysis were excluded from this study because their treatments were more complex than others and always need to conduct drug-eluting beads bronchial arterial chemoembolization (DEB-BACE), which can bias the outcome evaluation. The Ethics Committee of our hospital approved this study, and written informed consents were obtained from all patients.

### Bronchial artery embolization procedures

After admission, routine treatments (such as oxygen inhalation, electrocardiogram monitoring and hemostasis drugs) were given to the patients if necessary. Routine examinations (such as computerized tomography (CT) and bronchoscopy) were carried out to confirm etiology. Before BAE, all patients received CT bronchography and angiography (CTBA) to identify offending vessels of hemoptysis as follows: all CTBA procedures were performed in Digital Subtraction Angiography (DSA) room with the use of UNIQ Clarity FD20 (Philips Amsterdam, Netherlands) and nonionic contrast medium (Visipaque, 320 mg I/mL, GE, Ireland). Under the guidance of DSA, a 5F Cobra (Cordis, USA) catheter was selectively catheterized into the offending vessels at different angles using a modified Seldinger technique. The contrast medium was injected manually to offending vessels, then the direction and angle of the offending vessels in tangent position were fully displayed for next BAE procedures. The BAE procedures were performed using coaxial microcatheter technology under the guidance of DSA. A 2.7 F microcatheter (Terumo, Japan) was super-selectively catheterized into distance of offending vessels under the guidance of micro-guide wire. After the offending vessels were confirmed by DSA once again, the offending vessels were embolized by PVA (300–500 μm, Cook Inc., USA) or microspheres (300–500 μm, Jiangsu Hengrui Medicine Co. Ltd., China) (shown in Additional file 1: Figure S1).

### Technical success and clinical success assessment

Technical success was defined as the complete embolization of all offending vessels (including bronchial artery and non-bronchial systemic artery) [19]. Clinical success was defined as cessation of hemoptysis or reduction of hemoptysis volume > 50% during hospitalization after BAE [20].

### Follow-up

At the period of hospitalization, all patients were daily followed up to monitor hemoptysis status and adverse events. After discharging from hospital, regular follow-up was conducted by telephone or clinical visit. During follow-up, the hemoptysis status, survival status and adverse events were recorded. Hemoptysis-free survival was calculated from the date of BAE to the date of hemoptysis recurrence or death. Overall survival was calculated from the date of BAE to the date of death.

### Statistical analysis

Statistical analysis was conducted using SPSS 24.0 statistical software (IBM, USA). Figures were plotted using GraphPad Prism 7.01 software (GraphPad lnc., USA). According to the embolic materials, all patients were classified as PVA group (N = 90) and microspheres group (N = 62).

The differences of clinical features between two groups were determined by Student’s *t* test, Chi-square test, Fisher’s exact test or Wilcoxon rank sum test. The differences of technical success rate, clinical success rate, hemoptysis recurrence rate, mortality and adverse events occurrence rate between two groups were determined by Chi-square test or Fisher’s exact test. Hemoptysis-free survival and overall survival were displayed by Kaplan–Meier curve, and the differences of hemoptysis-free survival and overall survival between two groups were analyzed by log-rank test. Multivariate Cox’s proportional hazard regression was used to analyze hemoptysis-free survival and overall survival. *P* value < 0.05 was considered as statistically significant.

## Results

### Clinical characteristics of patients with hemoptysis

In microspheres group, the mean age was 61.8 ± 10.4 years, and the numbers of females as well as males were 21 (33.9%) and 41 (66.1%), respectively. As for in PVA group, the mean age was 59.5 ± 12.9 years, meanwhile, the numbers of females and males were 38 (42.2%) and 52 (57.8%) respectively (Table 1). No difference of age, gender, history of smoking, history of drinking, etiology, comorbidities, hemoptysis volume, or offending vessels was observed between microspheres group and PVA group (all *P* > 0.05). More detailed information about the clinical characteristics were shown in Table 1.

**Table 1:** Clinical features

Items	PVA group (N = 90)	Microspheres group (N = 62)	*P* value
Demographic characteristics			
Age (years), mean ± SD	59.5 ± 12.9	61.8 ± 10.4	0.243
Gender, No. (%)			0.299
Female	38 (42.2)	21 (33.9)	
Male	52 (57.8)	41 (66.1)	
History of smoking, No. (%)	34 (37.8)	30 (48.4)	0.193
History of drinking, No. (%)	25 (27.8)	20 (32.3)	0.552
Etiology, No. (%)			0.090
Bronchiectasis	81 (90.0)	49 (79.1)	
Tuberculosis	4 (4.5)	4 (6.5)	
NTM infection	2 (2.2)	3 (4.8)	
BA malformation	0 (0.0)	3 (4.8)	
Benign tumor of bronchus	1 (1.1)	0 (0.0)	
Diffuse lesions in bilateral lungs	1 (1.1)	0 (0.0)	
Aortic dissection stent implantation	1 (1.1)	0 (0.0)	
Unknown	0 (0.0)	3 (4.8)	
Comorbidities, No. (%)			
Hypertension	14 (15.6)	10 (16.1)	0.924
COPD	3 (3.3)	4 (6.5)	0.367
CHD	5 (5.6)	0 (0.0)	0.080
Chronic bronchitis	6 (6.7)	0 (0.0)	0.082
Hepatitis	4 (4.4)	1 (1.6)	0.649
Cerebral infarction	3 (3.3)	3 (4.8)	0.688
Diabetes mellitus	1 (1.1)	3 (4.8)	0.305
Hemoptysis volume, No. (%)			0.339
< 100 mL	63 (70.0)	46 (74.2)	
100–200 mL	8 (8.9)	11 (17.7)	
> 200 mL	19 (21.1)	5 (8.1)	
Offending vessels, No. (%)			0.682
Bilateral BA	59 (65.6)	34 (54.9)	
Bilateral BA + others^a^	16 (17.8)	16 (25.8)	
Right BA	9 (10.0)	7 (11.3)	
Right BA + others^b^	2 (2.2)	3 (4.8)	
Left BA	1 (1.1)	1 (1.6)	
Left BA + others^c^	3 (3.3)	1 (1.6)	

Comparison was determined by Student’s t test, Chi-square test, Fisher’s exact test or Wilcoxon rank sum test

*PVA* polyvinyl alcohol, *SD* standard deviation, *NTM* nontuberculous mycobacterium, *BA* bronchial artery, *COPD* chronic obstructive pulmonary disease, *CHD* coronary heart disease, *IPA* inferior phrenic artery, *ICA* intercostal artery, *IMA* internal mammary artery, *TTA* thyrocervical trunk artery, *CTA* costocervical trunk artery

^a^Others included IPA, ICA, IMA, TTA or CTA

^b^Others included ICA, IMA or TTA

^c^Others included IMA, IPA or ICA

### Comparison of success rate, hemoptysis recurrence rate and mortality

**Fig. 1:**
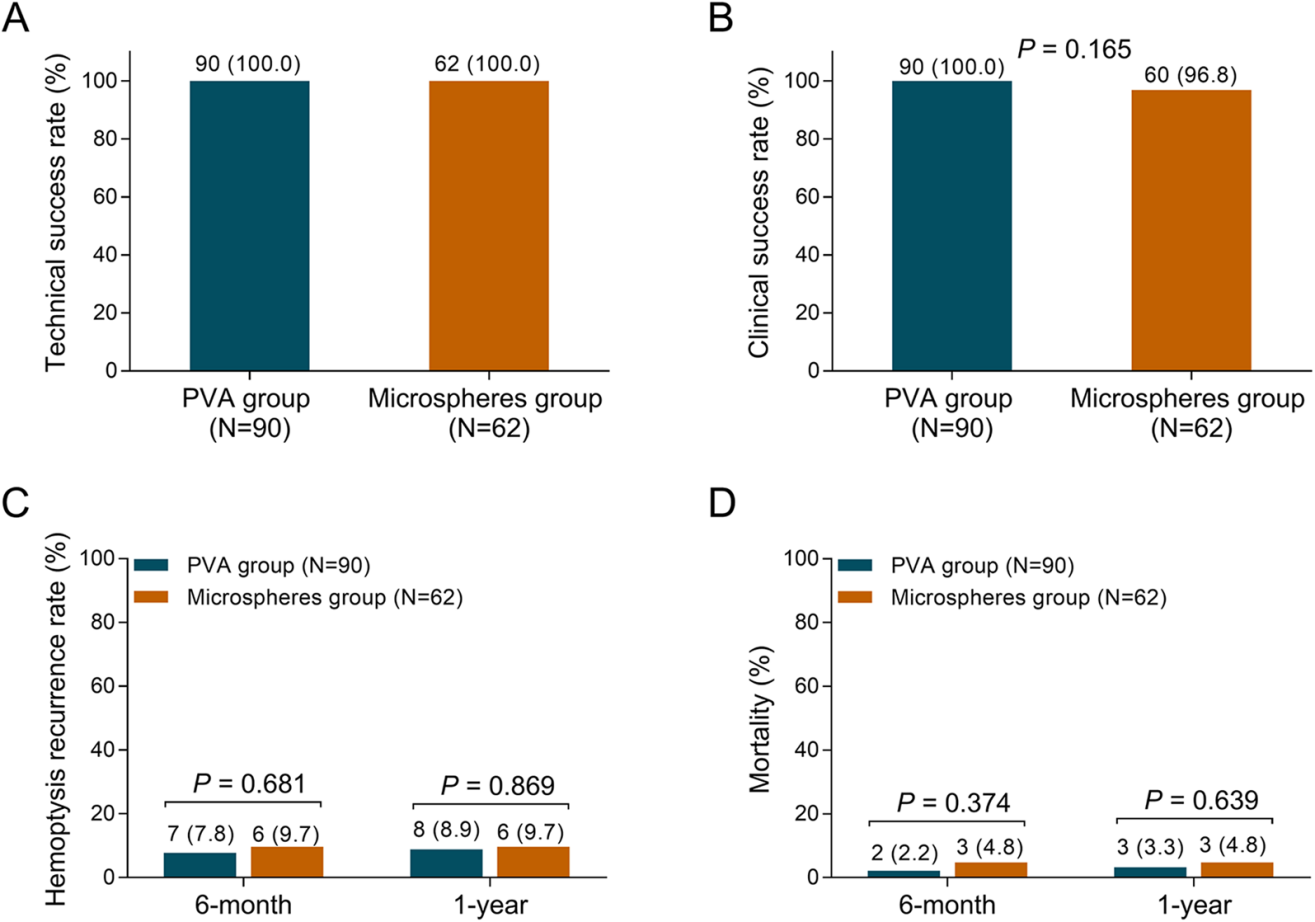
Technical success, clinical success, hemoptysis recurrence rates and mortality. Comparison of technical success rate (**A**), clinical success rate (**B**), hemoptysis recurrence rate (**C**) and mortality (**D**) between microspheres group and PVA group. PVA, polyvinyl alcohol

Technical success rates were both 100.0% in microspheres group and PVA group (Fig. 1A). Meanwhile, clinical success rate was of no difference between microspheres group (96.8%) and PVA group (100.0%) (*P* = 0.165) (Fig. 1B). Furthermore, microspheres group presented similar 6-month (9.7% vs. 7.8%, *P* = 0.681) and 1-year (9.7% vs*.* 8.9%, *P* = 0.869) hemoptysis recurrence rate compared to PVA group (Fig. 1C). As for morality, microspheres group exhibited similar 6-month (4.8% vs. 2.2%, *P* = 0.374) and 1-year (4.8% vs*.* 3.3%, *P* = 0.639) mortality compared with PVA group (Fig. 1D). These data was validated by further multivariate logistic regression analysis that group (microspheres vs. PVA) was not correlated with hemoptysis recurrence risk (*P* = 0.669) (Table 2) and mortality risk (*P* = 0.693) (Table 3). Meanwhile, only hemoptysis volume (OR = 2.205, *P* = 0.016) and offending vessels of left bronchial artery (vs. bilateral bronchial artery) (OR = 16.769, *P* = 0.016) were independent predictive factors for increased hemoptysis recurrence risk or mortality risk (Tables 2, 3).

**Table 2:** Multivariate logistic regression analysis of hemoptysis recurrence risk

Items	Multivariate logistic regression			
	*P* value	OR	95% CI	
			Lower	Higher
Group (microspheres vs. PVA)	0.669	1.273	0.421	3.847
Age (> 60 years)	0.481	0.679	0.232	1.990
Male	0.371	1.958	0.450	8.526
History of smoke	0.713	0.751	0.163	3.452
History of drink	0.480	1.665	0.404	6.860
Etiology (bronchiectasis vs. others)	0.673	0.911	0.589	1.408
Comorbidities (yes vs. no)	0.698	0.803	0.265	2.437
Hemoptysis volume	0.016	2.205	1.160	4.194
Offending vessels				
Bilateral BA	Reference	–	–	–
Left BA	0.131	4.410	0.644	30.220
Right BA	0.205	0.246	0.028	2.154

*OR* odds ratio, *CI* confidence interval, PVA polyvinyl alcohol, *BA* bronchial artery

**Table 3:** Multivariate logistic regression analysis of mortality risk

Items	Multivariate logistic regression			
	*P* value	OR	95% CI	
			Lower	Higher
Group (microspheres vs. PVA)	0.693	0.724	0.146	3.587
Age (> 60 years)	0.055	10.162	0.954	108.189
Male	0.884	1.162	0.154	8.738
History of smoke	0.918	0.892	0.103	7.733
History of drink	0.724	0.691	0.089	5.391
Etiology (bronchiectasis vs. others)	0.234	1.267	0.858	1.871
Comorbidities (yes vs. no)	0.918	0.920	0.188	4.498
Hemoptysis volume	0.852	0.902	0.305	2.669
Offending vessels				
Bilateral BA	Reference	–	–	–
Left BA	0.016	16.769	1.702	165.199
Right BA	0.710	1.541	0.158	15.045

*OR* odds ratio, *CI* confidence interval, *PVA* polyvinyl alcohol, *NTM* nontuberculous mycobacterium, *BA* bronchial artery

### Comparison of hemoptysis-free survival and overall survival

**Fig. 2:**
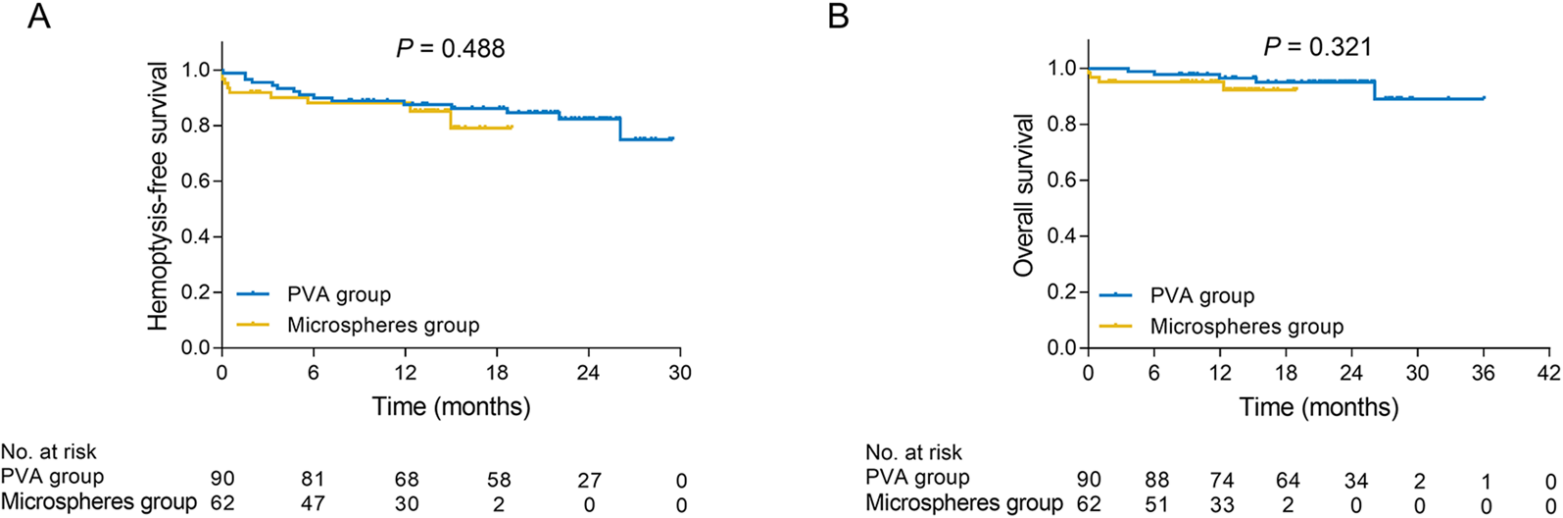
Survival analysis. Comparison of hemoptysis-free survival (**A**) and overall survival (**B**) between microspheres group and PVA group. PVA, polyvinyl alcohol

During the follow-up, the hemoptysis status and survival status were recorded and analyzed, which observed that microspheres group presented no difference of hemoptysis-free survival (*P* = 0.488) (Fig. 2A) or overall survival (*P* = 0.321) (Fig. 2B) compared with PVA group, which was further validated by multivariate Cox’s regression analysis (Tables 4, 5).

**Table 4:** Multivariate Cox’s regression analysis of hemoptysis-free survival

Items	Multivariate Cox’s proportional hazard regression			
	*P* value	HR	95% CI	
			Lower	Higher
Group (microspheres vs. PVA)	0.498	1.360	0.558	3.315
Age (> 60 years)	0.418	1.434	0.600	3.431
Male	0.507	1.469	0.471	4.581
History of smoke	0.973	0.980	0.296	3.241
History of drink	0.878	0.917	0.304	2.765
Etiology (bronchiectasis vs. others)	0.642	0.765	0.247	2.372
Comorbidities (yes vs. no)	0.840	0.914	0.384	2.179
Hemoptysis volume	0.145	1.454	0.879	2.405
Offending vessels				
Bilateral BA	Reference	–	–	–
Left BA	0.055	3.519	0.973	12.731
Right BA	0.457	0.569	0.129	2.507

*HR* hazards ratio, *CI* confidence interval, *PVA* polyvinyl alcohol, *NTM* nontuberculous mycobacterium, *BA* bronchial artery

**Table 5:** Multivariate Cox’s regression analysis of overall survival

Items	Multivariate Cox’s proportional hazard regression			
	*P* value	HR	95% CI	
			Lower	Higher
Group (microspheres vs. PVA)	0.570	1.533	0.350	6.713
Age (> 60 years)	0.066	7.373	0.878	61.906
Male	0.761	1.324	0.217	8.079
History of smoke	0.912	0.890	0.111	7.128
History of drink	0.609	0.588	0.077	4.494
Etiology (bronchiectasis vs. others)	0.825	0.782	0.088	6.927
Comorbidities (yes vs. no)	0.989	0.990	0.226	4.326
Hemoptysis volume	0.777	0.860	0.304	2.435
Offending vessels				
Bilateral BA	Reference	–	–	–
Left BA	0.097	4.792	0.754	30.446
Right BA	0.739	1.459	0.159	13.423

*HR* hazards ratio, *CI* confidence interval, *PVA* polyvinyl alcohol, *NTM* nontuberculous mycobacterium, *BA* bronchial artery

### Comparison of adverse events

All the adverse event occurred in microspheres group or PVA group were common and well tolerable (Table 6). In detail, there was no difference of cough/expectoration (*P* = 0.394), fever (*P* = 0.071), chest discomfort (*P* = 1.000), nausea/vomiting (*P* = 0.514), abdominal pain (*P* = 1.000), poor appetite and fatigue (P = 1.000), ecchymosis at the puncture site (P = 0.408), or allergy and dyspnea (P = 1.000) between the two groups (Table 6). More detailed information was shown in Table 6.

**Table 6:** Adverse events in total patients (N = 152)

Items	PVA group (N = 90)	Microspheres group (N = 62)	*P* value
Cough/expectoration	29 (32.2)	16 (25.8)	0.394
Fever	4 (4.4)	8 (12.9)	0.071
Chest discomfort	5 (5.6)	3 (4.8)	1.000
Nausea/vomiting	2 (2.2)	0 (0.0)	0.514
Abdominal pain	1 (1.1)	0 (0.0)	1.000
Poor appetite and fatigue	1 (1.1)	0 (0.0)	1.000
Ecchymosis at the puncture site	0 (0.0)	1 (1.6)	0.408
Allergy and dyspnea	1 (1.1)	0 (0.0)	1.000

Comparison was determined by Chi-square test or Fisher’s exact test

*PVA* polyvinyl alcohol

### Comparison of treatment efficacy and safety profiles in subgroup analysis

**Fig. 3:**
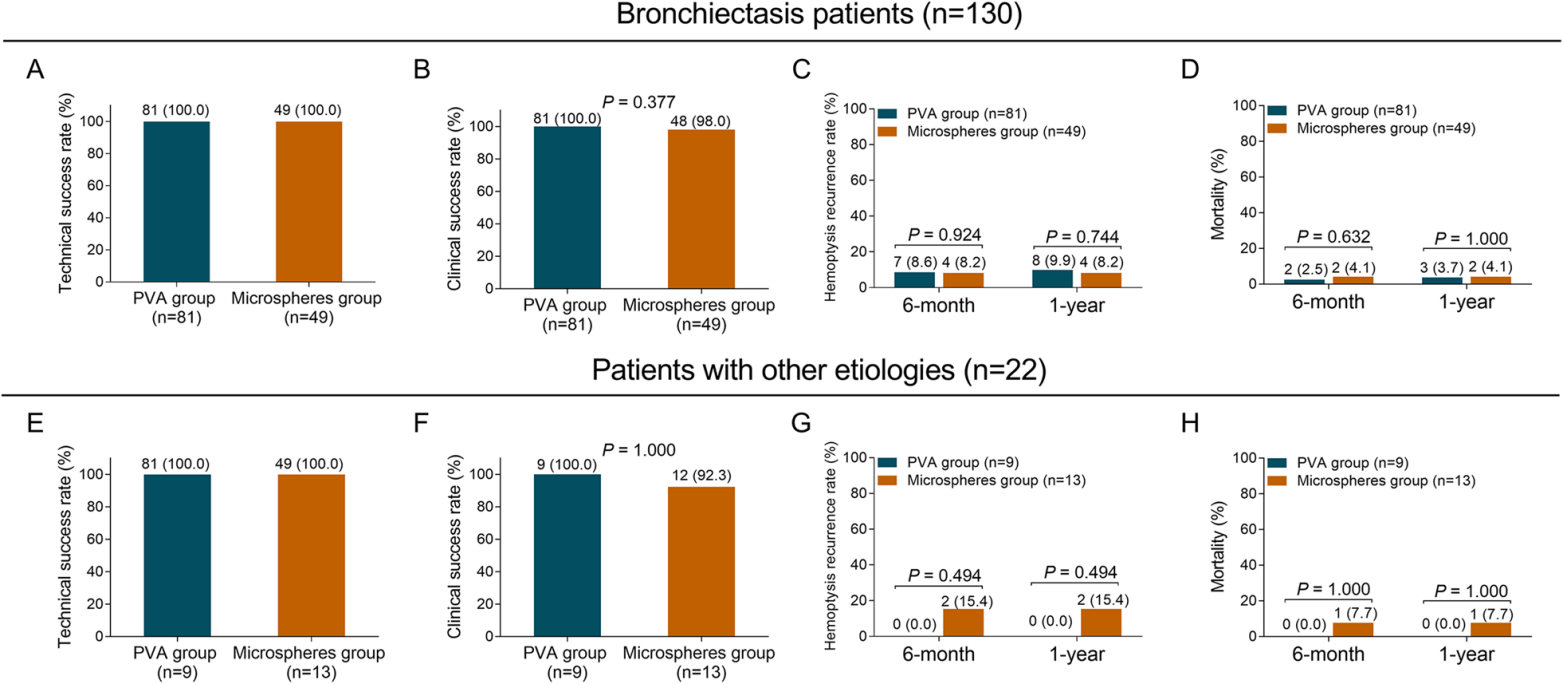
Technical success, clinical success, hemoptysis recurrence rates, and mortality in subgroup analyses. In bronchiectasis patients, comparison of technical success rate (**A**), clinical success rate (**B**), hemoptysis recurrence rate (**C**) and mortality (**D**) between microspheres group and PVA group. In patients with other etiologies, comparison of technical success rate (**E**), clinical success rate (**F**), hemoptysis recurrence rate (**G**) and mortality (**H**) between microspheres group and PVA group. PVA, polyvinyl alcohol

**Fig. 4:**
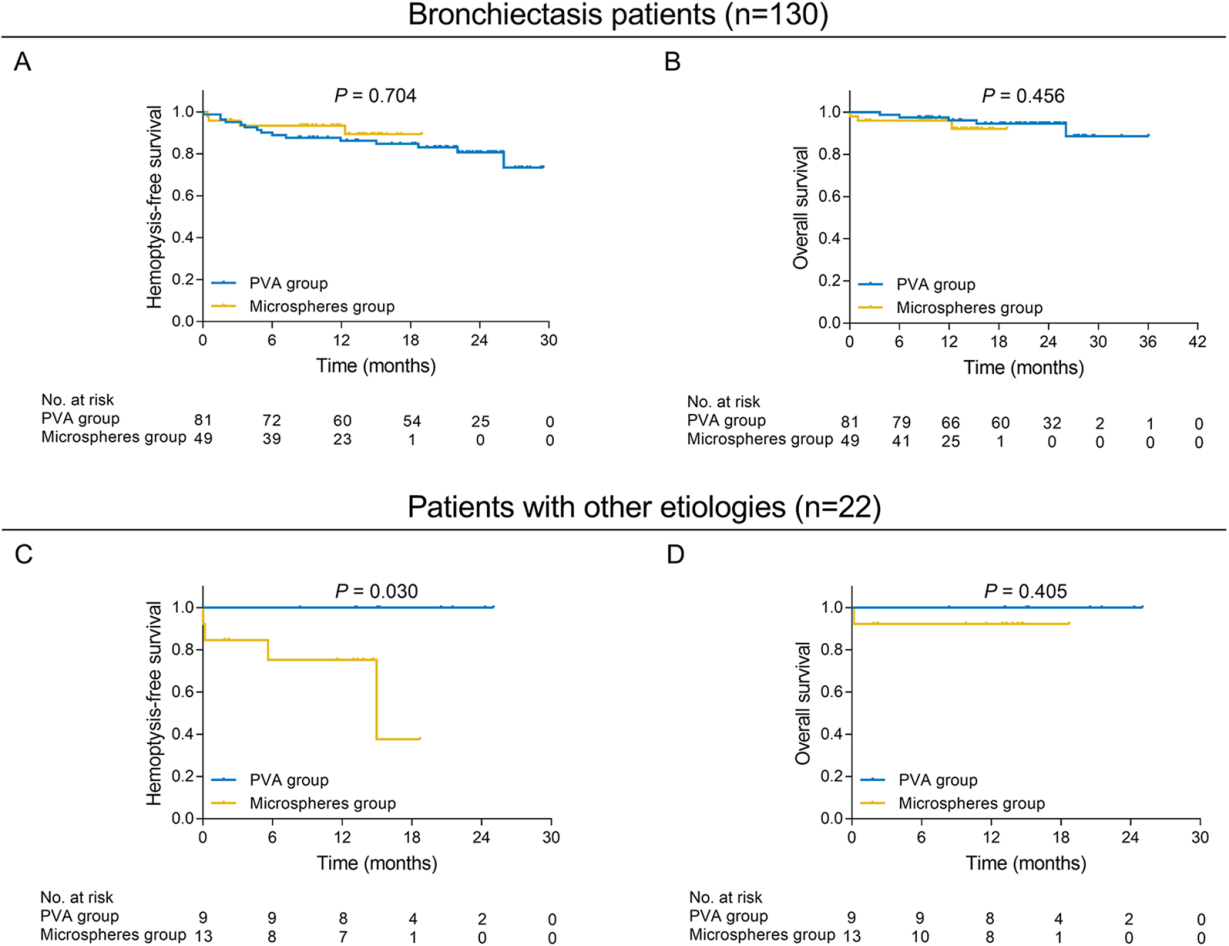
Survival in subgroup analyses. In bronchiectasis patients, comparison of hemoptysis-free survival (**A**) and overall survival (**B**) between microspheres group and PVA group. In patients with other etiologies, comparison of hemoptysis-free survival (**C**) and overall survival (**D**) between microspheres group and PVA group. PVA, polyvinyl alcohol

According to the hemoptysis etiology, patients were divided into two subgroups: bronchiectasis patients (n = 130) and patients with other etiologies (n = 22). In bronchiectasis patients, there was no difference of technical success rate, clinical success rate, hemoptysis recurrence rate, mortality, hemoptysis-free survival, overall survival, or adverse events between microspheres group and PVA group (all *P* > 0.05) (Fig. 3A–D, Fig. 4A, B, and Additional file 2: Table S1). In patients with other etiologies, there was also no difference of technical success rate, clinical success rate, hemoptysis recurrence rate, mortality, overall survival, or adverse events between microspheres group and PVA group (all *P* > 0.05), while hemoptysis-free survival was decreased in microspheres group compared with PVA group (*P* = 0.030) (Fig. 3E–H, Fig. 4C, D, and Additional file 2: Table S1).

## Discussion

Polyvinyl alcohol is one of the most widely used embolic agents for BAE as it presents several advantages including high cost-effective, diversity of embolic size depending on offending vessels, easy to handle, durable, etc., meanwhile, the efficacy of BAE with PVA in controlling hemoptysis has been illustrated by enormous evidence before [7, 9, 12, 14, 21]. For example, a retrospective study indicates that BAE with PVA is utilized in the treatment of 334 hemoptysis patients who presents with the most common etiology of pulmonary tuberculosis with technical success rate of 90.7%, and 6.5% procedures were repeated within two months due to technical or clinical failures [22]. Furthermore, another study exhibits that the immediate success rate is achieved in 86% patients, and during the 5-year follow-up, the recurrence rate of hemoptysis was 28% and mortality was 22% in patients who receives BAE using PVA for hemoptysis [23]. As for microspheres, they are compressible hydrogel with several advantages including out-standing biocompatibility, resistance to aggregate, satisfied elasticity, and are applied for the embolization in various arteriovenous, including uterine artery, prostatic artery, for treatment of several diseases [16, 17, 24, 25]. For example, microspheres exhibits greater ability in improving prostatic volume reduction and peak urinary flow compared with non-spherical PVA particles for prostatic artery embolization treatment in patients with lower urinary tracts symptoms [25]. However, its application in BAE treatment for hemoptysis was limited, therefore we explored the treatment efficacy and safety profiles of BAE with microspheres in hemoptysis patients.

According to previous study, the technical and clinical success rates of BAE using PVA in patients with hemoptysis are reported to be 77–100% and 85–99% respectively [7, 14]. In our study, for BAE treatment with PVA, the technical success rate of 100% observed was within the reported range before, while the clinical success rate of 100% was a little above the reported rage before, which might be due to relatively small sample size [7, 14]. In addition, there was no difference of clinical success rate (96.8 vs. 100.0%) between microspheres and PVA groups, which suggested that microspheres presented similar immediate effect on controlling hemoptysis as PVA. The possible reasons might include that (1) The short-term good efficacy of BAE using PVA or microspheres in our study might be attributed to the CTBA evaluation prior to BAE as the regular protocol, which increased the accuracy of identification in the bleeding sources and underlying cause of hemorrhage [3, 4]; (2) Furthermore, the majority of patients presented with mild-to-moderate hemoptysis, thereby decreasing the risk of failure to cannulate the bronchial artery or to facilitate a stable catheter position, which led to technical success rate of both 100% as well as similar clinical success between two groups [5, 26]; (3) In addition, considering that microspheres and PVA were made of the same embolic material and of the same size, these two embolic agents therefore displayed similar technical and clinical success rate [14, 27]. Of note, we found that offending vessels of left bronchial artery rather than right bronchial artery (vs. bilateral bronchial artery) was also an independent predictive factor for increased hemoptysis recurrence risk or mortality risk, which might be explained by that different anatomical structure. Anatomically, left BA is narrower and longer than right BA, which might increase the failure to identify culprit vessels and therefore enhance hemoptysis recurrence risk and mortality risk [28]. Furthermore, interestingly, we found that hemoptysis volume were independent predictive factors for increased hemoptysis recurrence risk or mortality risk, which might be associated with the difficulty of bleed controlling and unstable hemodynamics, increasing hemoptysis recurrence risk [6].

In our study, 6-month (PVA vs. microspheres: 9.7% vs. 7.8%) and 1-year (PVA vs. microspheres: 9.7% vs. 8.9%) hemoptysis recurrence rates were similar between BAE using microspheres and BAE using PVA. The incidences of hemoptysis recurrence in our study were a little reduced compared with the data in previous studies, which reported that the hemoptysis-recurrence rate after BAE was estimated to be 10–29% [7, 26]. The possible explanations might include (1) the relatively short follow-up in our study, (2) the exclusion of the patients with lung cancer, which was correlated with a great high likelihood of bleeding recurrence [29, 30]. Furthermore, according to the previous evidence, PVA presented increased aggregation compared with microspheres, which might lead to premature embolization proximal to the intended vascular level, further contributing to higher risk of long-term hemoptysis recurrence rate [14]. However, we did not observe the increased hemoptysis recurrence rate in PVA compared with microspheres in our study, which might be due to relatively small sample size and insufficient follow-up. Furthermore, based on existing evidence, the main causes of recurrent hemoptysis consisted of incomplete embolization of the vessels, revascularization of the collateral circulation or progression of the underlying pulmonary disease rather than embolic agents used, which might explain that microspheres displayed similar hemoptysis recurrence rates as PVA [14]. In addition, 6-month and 1-year mortality were also similar between PVA and microspheres in hemoptysis patients. The possible reasons include that (1) considering the prior data, PVA presented similar hemoptysis recurrence rate compared to microspheres, thereby contributing to the similar hemoptysis-related mortality between PVA and microspheres as well; (2) Based on the previous study, the primary cause for mortality after the treatment of BAE was the underlying progression of disease, and meanwhile considering the similar mechanism and short-term treatment efficacy between PVA and microspheres, therefore, BAE using PVA presented similar mortality to BAE using microspheres. In addition, no correlation of embolic agents with hemoptysis recurrence, mortality, hemoptysis-free survival and overall survival was further validated in multivariate logistic regression analysis. Moreover, further subgroup analysis revealed that there was no difference of clinical success rate, hemoptysis recurrence rate, morality, overall survival between PVA and microspheres in subgroup of bronchiectasis patients and patients with other etiologies, while hemoptysis-free survival was increased in patients receiving BAE using PVA compared with patients receiving BAE using microspheres in subgroup of patients with other etiologies, which might be attributed to the relatively small sample size.

As for adverse events, there was no difference of adverse event incidence between BAE using PVA and BAE using microspheres, and main adverse events were relatively minor and well-tolerated (including cough/expectoration, fever, chest discomfort, nausea/vomiting, abdominal pain, poor appetite and fatigue, ecchymosis at the puncture site, and allergy as well as dyspnea). Theses minor adverse events were common in BAE procedures, which was in accordance with the observation in previous studies [31]. Possible explanations for the lack of major complications included that (1) The size of PVA and microspheres were both 300–500 μm in diameter, which could fit with pulmonary artery and avoid distal occlusion of normal peripheral branches, decreasing the possibility of some major complications (such as: esophageal, bronchial, pulmonary artery necrosis) caused by ischemia [7]; (2) Furthermore, with the help of routine CTBA evaluation before BAE combined with angiography during procedure, the spinal collaterals were clearly identified, which decreased the possibility of off-target embolization of the spinal artery as well as the risk of BAE-related neurologic complications [3]. Furthermore, considering the comparative cost-efficacy, microsphere (CNY1600/bottle) was cheaper than PVA (CNY1710/bottle), it was more reasonable to use microsphere rather than PVA.

Our study filled the gap to assess the efficacy and safety profile of Chinese local microspheres and further to compare its efficacy and safety with frequently-used PVA for BAE treatment in hemoptysis patients, which observed that microspheres presented similar efficacy on controlling hemoptysis and safety profile as PVA. Meanwhile, considering the more favorable cost of microspheres compared with PVA, the evidence suggested that microsphere might be an alternative option to PVA in BAE treatment. However, the present study still existed some limitations including (1) the sample size was relatively small in our study, which might lead to less statistical validation, more patients from multiple centers were needed for validation; (2) Longer follow-up was needed to observe the adverse events in hemoptysis patients underwent BAE; (3) The majority of patients included were with mild-to-moderate hemoptysis, therefore, more studies were necessary for extending our results in the treatment of massive hemoptysis [5, 26]; (4) As this present study was an observational study, the patients enrolled in our study were not randomized into two groups, which might contribute to selection bias and confounding factors in present study.

In conclusion, BAE with microspheres presents comparable efficacy and safety profiles compared with BAE with PVA for the treatment of hemoptysis, therefore, microspheres may serve as an alternative embolic agent in hemoptysis management.

## Supplementary Information

**Additional file1. **Technical success, clinical success, hemoptysis recurrence rates and mortality

**Additional file2:** **Table S1.** Subgroup analysis of adverse events.

## Data Availability

Data sharing is not applicable to this article as no datasets were generated or analysed during the current study.
