# Supplementary figures and images for: Microspheres present comparable efficacy and safety profiles compared with polyvinyl alcohol for bronchial artery embolization treatment in hemoptysis patients
Source: J Transl Med. 2021 Oct 11;19:422. doi: 10.1186/s12967-021-02947-7 (PMC8504013; doi:10.1186/s12967-021-02947-7)

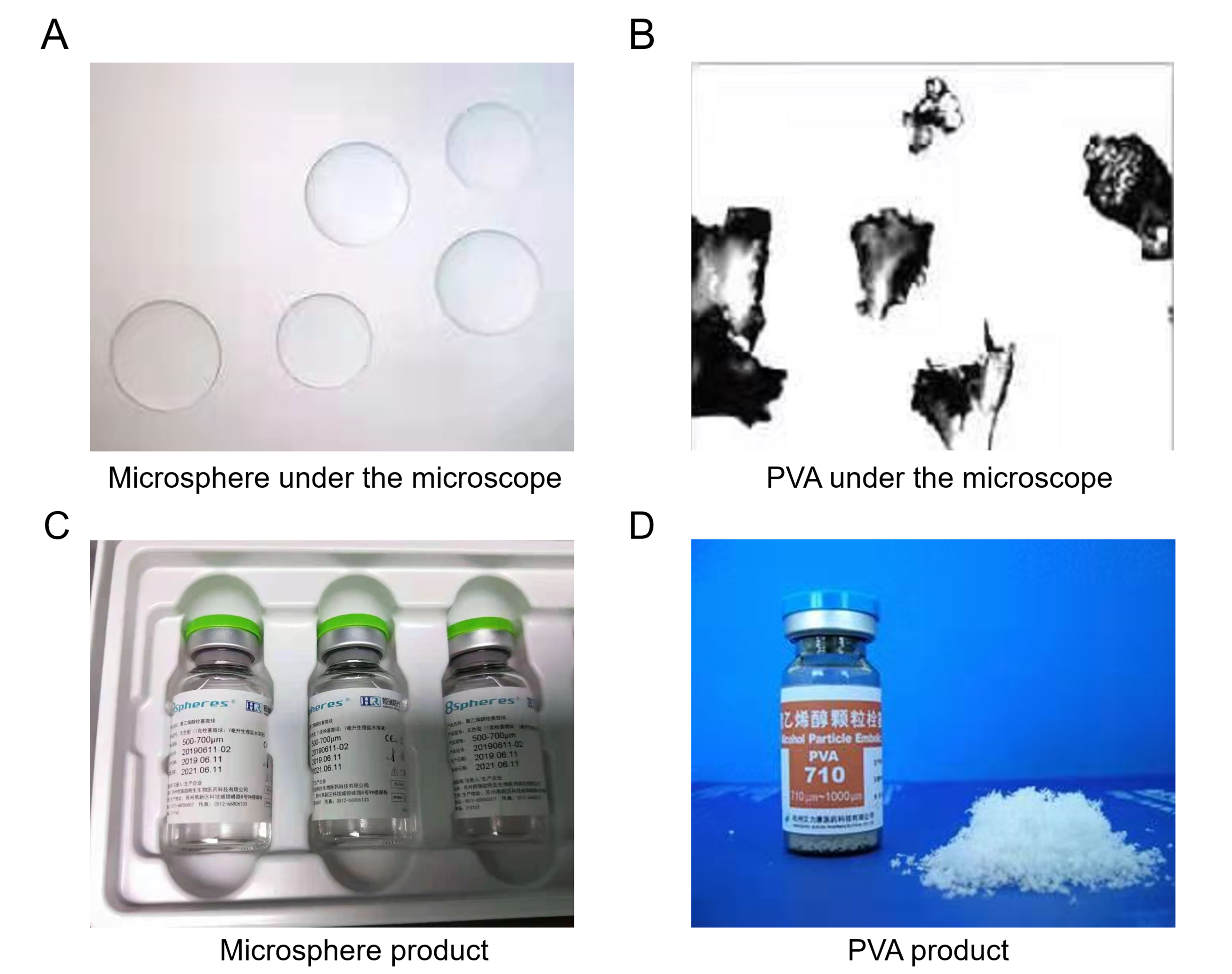

Supplement: Supplementary file 1 — Additional file1. Technical success, clinical success, hemoptysis recurrence rates and mortality [file 12967_2021_2947_MOESM1_ESM.tif]
