# Supplementary material for: Microspheres present comparable efficacy and safety profiles compared with polyvinyl alcohol for bronchial artery embolization treatment in hemoptysis patients
Source: J Transl Med. 2021 Oct 11;19:422. doi: 10.1186/s12967-021-02947-7 (PMC8504013; doi:10.1186/s12967-021-02947-7)
Supplement: Supplementary file 2 — Additional file2: Table S1. Subgroup analysis of adverse events. [file 12967_2021_2947_MOESM2_ESM.docx]

**Supplementary Table 1.** Subgroup analysis of adverse events

| Items | Bronchiectasis patients (n=130) | | | Patients with other etiologies (n=22) | | |
| --- | --- | --- | --- | --- | --- | --- |
|  | PVA group (n=81) | Microspheres group (n=49) | *P* value | PVA group (n=9) | Microspheres group (n=13) | *P* value |
| Cough/expectoration | 24 (29.6) | 13 (26.5) | 0.704 | 5 (55.6) | 3 (23.1) | 0.187 |
| Fever | 4 (4.9) | 6 (12.2) | 0.130 | 0 (0.0) | 2 (15.4) | 0.494 |
| Cheat discomfort | 5 (6.2) | 2 (4.1) | 0.609 | 0 (0.0) | 1 (7.7) | 1.000 |
| Nausea/vomiting | 2 (2.5) | 0 (0.0) | 0.268 | 0 (0.0) | 0 (0.0) | - |
| Abdominal pain | 1 (1.2) | 0 (0.0) | 1.000 | 0 (0.0) | 0 (0.0) | - |
| Poor appetite and fatigue | 1 (1.2) | 0 (0.0) | 1.000 | 0 (0.0) | 0 (0.0) | - |
| Ecchymosis at the puncture site | 0 (0.0) | 0 (0.0) | - | 0 (0.0) | 1 (7.7) | 1.000 |
| Allergy and dyspnea | 1 (1.2) | 0 (0.0) | 1.000 | 0 (0.0) | 0 (0.0) | - |

Comparison was determined by Chi-square test or Fisher's exact test. PVA, polyvinyl alcohol.
